# A biotechnology perspective of fungal proteases

**DOI:** 10.1590/S1517-838246220140359

**Published:** 2015-06-01

**Authors:** Paula Monteiro de Souza, Mona Lisa de Assis Bittencourt, Carolina Canielles Caprara, Marcela de Freitas, Renata Paula Coppini de Almeida, Dâmaris Silveira, Yris Maria Fonseca, Edivaldo Ximenes Ferreira, Adalberto Pessoa, Pérola Oliveira Magalhães

**Affiliations:** 1 Universidade de São Paulo, Departamento de Tecnologia Bioquimico-Farmacêutica, Faculdade de Ciências Farmacêuticas, Universidade de São Paulo, São Paulo, SP, Brasil, Departamento de Tecnologia Bioquimico-Farmacêutica, Faculdade de Ciências Farmacêuticas, Universidade de São Paulo, São Paulo, SP, Brazil.; 2 Universidade de Brasília, Departamento de Farmácia, Faculdade de Ciências da Saúde, Universidade de Brasília, Brasília, DF, Brasil, Departamento de Farmácia, Faculdade de Ciências da Saúde, Universidade de Brasília, Brasília, DF, Brazil.; 3 Universidade de Brasília, Laboratório de Enzimologia, Departamento de Biologia Celular, Universidade de Brasília, Brasília, DF, Brasil, Laboratório de Enzimologia, Departamento de Biologia Celular, Universidade de Brasília, Brasília, DF, Brazil.

**Keywords:** proteases, enzyme production, fungal protease, industrial application

## Abstract

Proteases hydrolyze the peptide bonds of proteins into peptides and amino acids, being found in all living organisms, and are essential for cell growth and differentiation. Proteolytic enzymes have potential application in a wide number of industrial processes such as food, laundry detergent and pharmaceutical. Proteases from microbial sources have dominated applications in industrial sectors. Fungal proteases are used for hydrolyzing protein and other components of soy beans and wheat in soy sauce production. Proteases can be produced in large quantities in a short time by established methods of fermentation. The parameters such as variation in C/N ratio, presence of some sugars, besides several other physical factors are important in the development of fermentation process. Proteases of fungal origin can be produced cost effectively, have an advantage faster production, the ease with which the enzymes can be modified and mycelium can be easily removed by filtration. The production of proteases has been carried out using submerged fermentation, but conditions in solid state fermentation lead to several potential advantages for the production of fungal enzymes. This review focuses on the production of fungal proteases, their distribution, structural-functional aspects, physical and chemical parameters, and the use of these enzymes in industrial applications.

## Introduction

Microbial proteases are among the most important hydrolytic enzymes and have been studied extensively. Proteases from microorganisms have attracted a great deal of attention in the last decade because of their biotechnology potential in various industrial processes such as detergent, textile, leather, dairy and pharmaceutical preparations. Although proteolytic enzymes from microorganisms are the source preferred in industrial application of enzymes due to the technical and economic advantage (Saran *et al.*, 2007). Microbial proteases are among the most important hydrolytic enzymes and have been studied extensively. This group represents one of the largest groups of industrial enzymes and accounts for approximately 60% of the total enzyme sales in the world (Zambare *et al.*, 2011). Fungal proteases have attracted the attention of environmental biotechnologists because fungi can grow on low cost substrates and secrete large amount of enzymes into culture medium which could ease downstream processing (Anitha and Palanivelu, 2013). In this present review, some aspects of fungal proteolytic enzymes are discussed, with reference to the production of protease, their distribution and their industrial applications.

## Proteases

Proteases (peptidases or proteolytic enzymes) constitute a large group of enzymes that catalyse the hydrolysis of peptide bonds in other proteins. Cleavage of peptide bonds lead to degradation of protein substrates into their constituent amino acids, or it can be specific, leading to selective protein cleavage for post-translational modification and processing. Proteases are classified as peptide hydrolases or peptidases (EC 3.4) and constitute a large family of enzymes, divided into endopeptidases (EC 3.4.21-99) and exopeptidases (EC 3.4.11-19), classified according to the position of the peptide bond to be cleaved. They can also be classified according to the pH range which they have a higher activity: acidic (pH 2.0 to 6.0), neutral (pH 6.0 to 8.0) and alkaline (pH 8.0 to 13.0) (Gupta *et al.*, 2002; Rao *et al.*, 1998; Sabotic and Kos, 2012).

The proteolytic enzymes are subdivided into two major groups, exopeptidases and endopeptidases, depending on their site of action. The exopeptidases act only near the ends of polypeptide chains. Based on their site of action at the N or C terminus, they are classified as amino and carboxypeptidases. Aminopeptidases (EC 3.4.14) act at a free N terminus of the polypeptide chain and liberate a single amino acid residue, a dipeptide, or a tripeptide. And the carboxypeptidases act at C terminals of the polypeptide chain and liberate a single amino acid or a dipeptide. Carboxypeptidases can be divided into three major groups, serine peptidases (EC 2.4.16), metallopeptidases (EC 2.4.17), and cysteine peptidases (EC 2.4.18), based on the nature of the amino acid residues at the active site of the enzymes. Endopeptidases are characterized by their preferential action at the peptide bonds in the inner regions of the polypeptide chain. The endopeptidases are divided into four subgroups based on their catalytic mechanism, serine proteases (EC 2.4.21), cysteine proteases (EC 2.4.22), aspartic proteases (EC 2.4.23), metalloproteases (EC 2.4.24) (Rao *et al.*, 1998).

Serine proteases are characterized by the presence of a serine group in their active site. They are generally active at neutral and alkaline pH, with optima at pH 7–11, low molecular mass (18–35 kDa) and have applications in a number of industries (Gupta *et al.*, 2002). Aspartic acid proteases, commonly known as acidic proteases, are the endopeptidases that depend on aspartic acid residues for their catalytic activity. The activity of all cysteine proteases depends on a catalytic dyad consisting of cysteine and histidine. Generally, cysteine proteases are active only in the presence of reducing agents such as HCN or cysteine. Papain is the best-known cysteine protease. Metalloproteases are the most diverse of the catalytic types of proteases. They are characterized by the requirement for a divalent metal ion for their activity (Rao *et al.*, 1998; Vranova *et al.*, 2013).

Proteases occur in animals, plants and microorganism, and have critical role in many physiological and pathological processes such as protein catabolism, blood coagulation, cell growth and migration, tissue arrangement, morphogenesis in development, inflammation, tumor growth and metastasis, activation of zymogens, release of hormones and pharmacologically active peptides from precursor proteins, and transport of secretory proteins across membranes (Rao *et al.*, 1998). Extracellular proteases catalyse the hydrolysis of proteins into smaller peptides and amino acids for subsequent absorption into cells, constituting a very important step in nitrogen metabolism (Sabotic and Kos, 2012).

## Protease Production

Proteases can be cultured in large quantities in a relatively short time by established methods of fermentation and they also produce an abundant, regular supply of the desired product (Gupta *et al.*, 2002). In general, microbial proteases are extracellular in nature and are directly secreted into the fermentation broth by the producer, thus simplifying downstream processing of the enzyme as compared to proteases obtained from plants and animals (Savitha *et al.*, 2011).

Microorganisms elaborate a large array of proteases, which are intracellular and extracellular. Intracellular proteases are important for various cellular and metabolic processes, such as sporulation and differentiation, protein turnover, maturation of enzymes and hormones and maintenance of the cellular protein pool. Extracellular proteases are important for the hydrolysis of proteins in cell-free environments and enable the cell to absorb and utilize hydrolytic products (Gupta *et al.*, 2002).

On an industrial scale, exoproteases are produced in complex media containing glucose and other costly substrates. Cultivation conditions are essential in successful enzyme production, that's why optimization of parameters such as pH, temperature and media composition must be controlled in process development (Abidi *et al.*, 2011). Specifically, the protease production is mainly influenced by the variation in C/N ratio, presence of some easily metabolizable sugars, such as glucose, and metal ion, besides several other physical factors, such as aeration, inoculum density, pH, temperature and incubation time. Protease synthesis is also affected by rapidly metabolizable nitrogen sources, such as amino acids in the medium (Gupta *et al.*, 2002). For improving protease production, have been used screening for hyper-producing strains, cloning and over-expression, controlled batch and fed-batch fermentations using simultaneous control of glucose, ammonium ion concentration, oxygen tension, pH and salt availability, chemostat fermentations, and optimization of the fermentation medium through a statistical approach, such as response surface methodology (Gupta *et al.*, 2002).

Filamentous fungi are used in many industrial processes for the production of enzymes and metabolites. Among the many advantages offered by the production of enzymes by fungi are low material costs coupled with high productivity, faster production, and the ease with which the enzymes can be modified. Further, the enzymes, being normally extracellular, are easily recoverable from the media (Vishwanatha *et al.*, 2010b). Proteases production of fungal origin have an advantage over bacterial protease as mycelium can be easily removed by filtration. Besides, the use of fungi as enzyme producer is safer than the use of bacteria, since they are normally recognised as GRAS (generally regarded as safe) (Germano *et al.*, 2003).

Proteases have been produced in submerged (SmF) and solid-state fermentations (SSF), and each technique has particular advantages the other unable to match (Sandhya *et al.*, 2005; Sun and Xu, 2009). SSF has certain advantages over the conventional SmF, like low production cost, uses raw materials as substrates, requires less energy and space, encounters less problems in downstream processing, stability of the product due to less dilution in the medium, and manufactures with higher productivity (Das and Mukherjee, 2007; Sun and Xu, 2009). SmF has advantages in process control and easy recovery of extracellular enzymes, mycelia or spores. However, the products are dilute and enzymatic extracts might be less stable than those from SSF. The major problems in large-scale SSF for fungal growth are the limited water and heat removal. In SmF, water is abundantly present and variations on temperature, oxygen concentration and nutrients are small (Biesebeke *et al.*, 2002). In addition, the minimal amount of water allows the production of metabolites in a more concentrated form, making the downstream processing less time consuming and less expensive. However, the conditions in SSF, especially the low moisture content in the system, lead to several potential advantages for the production of fungal enzymes. Firstly, these conditions favour the growth of filamentous fungi, which typically grow in nature on solid substrates, such as pieces of wood, leaves and roots of plants and other organic natural materials. Secondly, the low moisture content can minimize problems with bacterial contamination during the fermentation. Finally, the environmental conditions in solid-state fermentation can stimulate the microorganism to produce enzymes with different properties than those of enzymes produced by the same organism under the conditions experienced in submerged fermentation (Germano *et al.*, 2003).

Different mechanisms have been described to regulate the synthesis and secretion of extracellular protease. The presence of a substrate can induce protease secretion. High levels of end products, such as amino acids, NH^+^_4_ and easily metabolizable sources of carbon may repress production. On the other hand, protease production may be increased when insufficient levels of carbon, nitrogen or sulfur are available. Finally, extracellular enzymes may be secreted constitutively at low levels regardless of the availability of a substrate (Geisseler and Horwath, 2008). The production of proteases by microorganisms is known to be influenced by the quality of the nitrogen source. Although complex nitrogen sources are usually used for protease production, the requirement for a specific nitrogen supplement differs from organism to organism. Generally, the fungi produce more proteolytic enzyme on a more complex proteinaceous nitrogen sources than on low molecular weight or inorganic nitrogen sources (Kucera, 1981). Enzyme synthesis was found to be repressed by rapidly metabolizable nitrogen sources such as amino acids or ammonium ion concentrations in the medium (Kumar and Takagi, 1999).

## Fungal Proteases

In recent years, there have been attempts to produce different types of protease trough SmF or SSF, using several different types of substrates. A great number of fungal strains have been used to produce proteases belonging to the genera *Aspergillus*, *Penicillium*, *Rhizopus*, *Mucor*, *Humicola*, *Thermoascus*, *Thermomyces*, among others. The physical and chemical parameters of protease from fungi have been widely studied and described. Table 1 shows properties of some proteases from fungi. It is interesting to notice that although a number of substrates have been employed for cultivating different fungi, wheat bran has been the preferred choice in most of the studies.

**Table 1:** Properties of some fungal proteases.

Fungi	Fermentation	pH optimal/stability	Temperature optimal/stability	Total/specific activity (U)	Type of protease	Type of substrate	Reference
*Aspergillus* sp. 13.33	SSF	-	45 °C	844.6	-	Wheat bran	(Macchione *et al.*, 2008)
*Aspergillus* sp. 13.34	SSF	-	45 °C	469.6	-	Wheat bran	(Macchione *et al.*, 2008)
*Aspergillus* sp. 13.35	SSF	-	45 °C	640.5	-	Wheat bran	(Macchione *et al.*, 2008)
*Aspergillus* sp.	SSF	-	30 °C	107.66	-	Soybean	(Rajmalwar and Dabholkar, 2009)
*Aspergillus awamori*	SmF	5.0	55 °C	-	Acid protease	Wheat bran	(Negi and Banerjee, 2009)
*Aspergillus clavatus* ES1	SmF	8.5	50 °C	4970	Alkaline protease	Wheat bran, fish flour	(Hajji *et al.*, 2007)
*Aspergillus flavus* 1.2	SSF	-	45 °C	117.6	-	Wheat bran	(Macchione *et al.*, 2008)
*Aspergillus flavus*	SSF	7.5–9.5	32 °C	6.8	Alkaline protease	Wheat bran	(Malathi and Chakraborty, 1991)
*Aspergillus flavus*	SSF	7.5	45 °C	640	Alkaline serine	Wheat bran	(Kranthi *et al.*, 2012)
*Aspergillus flavus*	SSF	7.0	36 °C	1894	Alkaline protease	Wheat bran, soy protein	(Agrawal *et al.*, 2005)
*Aspergillus fumigates*	SSF	8.0	50 °C	-	Serine protease	Wheat bran	(Silva *et al.*, 2010)
*Aspergillus niger*	SmF	4.0	30 °C	200	Acid protease	Rice	(Yang and Lin, 1998)
*Aspergillus niger*	SmF	3.0	27 °C	3600	-	Yeast extract, malt extract, peptone and dextrose	(O'Donnell *et al.*, 2001)
*Aspergillus oryzae* IAM2704	SmF	7.0	30 °C	1550	Neutral protease	Casein, glucose	(Ogawa *et al.*, 1995)
*Aspergillus oryzae* MTCC 5341	SSF	3.0–4.0	55 °C	43.658	Aspartate protease	Wheat bran	(Vishwanatha *et al.*, 2009)
*Aspergillus oryzae* MTCC 5341	SSF	6.3	55 °C	3500	Aspartate protease	Wheat bran, Soy flour, Skim milk	(Vishwanatha *et al.*, 2010a)
*Aspergillus oryzae* NCIM 649	SSF	7.0	36 °C	6301	Alkaline protease	Wheat bran, soy protein	(Agrawal *et al.*, 2005)
*Aspergillus oryzae* NCIM 1212	SSF	7.0	36 °C	1631	Alkaline protease	Wheat bran, soy protein	(Agrawal *et al.*, 2005)
*Aspergillus oryzae* NCIM 1032	SSF	7.0	36 °C	4744	Alkaline protease	Wheat bran, soy protein	(Agrawal *et al.*, 2005)
*Aspergillus parasiticus*	SSF	8.0	40 °C	17.65	Serine protease	Wheat bran	(Tunga *et al.*, 2003)
*Aspergillus parasiticus*	SSF	7.0	36 °C	9545	Alkaline protease	Wheat bran, soy protein	(Agrawal *et al.*, 2005)
*Aspergillus ustus*	SmF	9.0	45 °C	1639	Serine protease	Skim milk	(Damare *et al.*, 2006)
*Beauveria* sp. MTCC 5184	SmF	6.5–7.5	28 °C	9783	Serine protease	Mustard seed cake	(Shankar *et al.*, 2011)
*Beauveria bassiana*	SmF	7.0	26 °C	280.72	Alkaline protease	Shrimp shell, soy powder	(Rao *et al.*, 2006)
*Beauveria felina*	SSF	7.0	28 °C	8211	Alkaline protease	Wheat bran, soy protein	(Agrawal *et al.*, 2005)
*Botrytis cinérea*	SmF	8.0	50 °C	388	Alkaline protease	Spirulina algae	(Abidi *et al.*, 2011)
*Clonostachys rosea*	SmF	9.0 – 10.0	60 °C	149.3	Serine protease	Glucose, gelatin, peptone, yeast extract	(Li *et al.*, 2006)
*Conidiobolus coronatus*	SmF	6.0 – 7.0	28 °C	72.46	Alkaline protease	Malt extract, glucose, yeast extract, petone	(Laxman *et al.*, 2005)
*Cordyceps militaris*	SmF	8.5 – 12.0	25 °C	818.7	Trypsin-like protease	Colloidal chitin	(Hattori *et al.*, 2005)
*Cordyceps sinensis*	SmF	7.0	40–55 °C	-	Chymotrypsin-like serine	Sucrose, mannose, galactose, tryptone, yeast extract	(Zhang *et al.*, 2008)
*Engyodontium album*	SSF	10.0 – 11.0	45–60 °C	3186	Alkaline protease	Wheat bran	(Chellappan *et al.*, 2006)
*Fusurium oxysporum*	SmF	8.0	45 °C	492	Trypsin-like protease	Gelatin	(Barata *et al.*, 2002)
*Graphium putredinis*	-	7.0	50 °C	114	Serine protease	Soya bean meal	(Savitha *et al.*, 2011)
*Hirsutella rhossiliensis*	SmF	7.0	40 °C	7.7	Serine protease	Panagrellus redivivus	(Wang *et al.*, 2007)
*Humicola lutea* 120-5	SmF	3.0 – 3.5	28 °C	1100	Acid proteinase	Glucose, casein	(Aleksieva and Peeva, 2000)
*Metarhizium anisopliae*	SmF	5.5	28 °C	1.12	-	Czapek-Dox	(Kucera, 1981)
*Metarhizium anisopliae* MTCC 892	SSF	7.0	28 °C	6452	Alkaline protease	Wheat bran, soy protein	(Agrawal *et al.*, 2005)
*Mucor circinelloides*	SmF	5.2	25 °C	25	-	Glucose	(Andrade *et al.*, 2002)
*Myceliophthora* sp.	SmF/SSF	7.0/9.0	50 °C	1.78	Alkaline protease	Casein, wheat bran	(Zanphorlin *et al.*, 2010)
*Ophiostoma piceae* 387N	-	7.0 – 9.0	40 °C	56.1	Subtilisin serine proteinase	Soybean	(Abraham and Breuil, 1996)
*Penicillium* sp.	SSF	6.0 – 9.0	45 °C	51.6	Serine protease	Defatted soybean	(Germano *et al.*, 2003)
*Penicillium* sp.	SSF	7.0	36 °C	4819	Alkaline protease	Wheat bran, soy protein	(Agrawal *et al.*, 2005)
*Penicillium camemberti*	SmF	3.4	50 °C	1276	Acid proteinase	-	(Chrzanowska *et al.*, 1995)
*Penicillium citrinum*	-	7.0	40 °C	-	Serine proteinase	-	(Yamamoto *et al.*, 1993)
*Penicillium griseoroseum* IH-02	SSF	5.0	30 °C	8.2	Acid protease	Soybean meal, wheat bran	(Ikram-Ul-Haq and Mukhtar, 2007)
*Penicillium restrictum*	SSF	7.0 – 8.0	37 °C	7.9	-	Starch	(Gombert *et al.*, 1999)
*Penicillium roqueforti*	-	5.0	25 °C	1394.7	Acid protease	Czapek-Dox, peptone	(Larsen *et al.*, 1998)
*Phanerochaete chrysosporium*	SSF	4.5	25 °C	35	Acid and thiolproteases	Glucose	(Cabaleiro *et al.*, 2002)
*Phanerochaete chrysosporium*	SmF	6.5	37 °C	231–290	-	Glucose	(Xiaoping *et al.*, 2008)
*Phanerochaete radiata*	SSF	4.5	25 °C	50	Thiolproteases	Glucose	(Cabaleiro *et al.*, 2002)
*Rhizomucor* sp.	SSF	-	45 °C	770.9	-	Wheat bran	(Macchione *et al.*, 2008)
*Rhizopus* SMC	SmF	4.3	28 °C	2000	Acid protease	Wheat bran, casein	(Ramamurthy *et al.*, 1991)
*Rhizopus oryzae*	SSF	5.5	60 °C	760	Aspartate protease	Wheat bran	(Kumar *et al.*, 2005)
*Thermoascus aurantiacus*	SSF	-	45 °C	258.3	-	Wheat bran	(Macchione *et al.*, 2008)
*Thermoascus aurantiacus*	SSF	5.5	60 °C	248	Acid protease	Wheat bran	(Merheb *et al.*, 2007)
*Thermomucor indicae-seudaticae* N31	SSF	5.7	70 °C	160	Acid protease	Wheat bran	(Merheb-Dini *et al.*, 2010)
*Thermomyces lanuginosus*	SmF	6.0	50 °C	0.71	-	Glucose, citric acid	(Jensen *et al.*, 2002)
*Thermomyces lanuginosus*	SSF	-	45 °C	945.2	-	Wheat bran	(Macchione *et al.*, 2008)
*Thermomyces lanuginosus*	SmF	5.0	70 °C	12.8	Serine protease	Casein, glucose, yeast extract	(Li *et al.*, 1997)
*Trichoderma harzianum*	-	8.0	60 °C	99	Serine protease	Soya bean meal	(Savitha *et al.*, 2011)
*Trichoderma reesei* QM9414	-	8.0	50 °C	0.25	Trypsin serine proteases	Glucose	(Dienes *et al.*, 2007)

The microbial proteases of *Aspergillus* species, in particular, have been studied in detail since they are known for their capacity to secrete high levels of enzymes in their growth environment. Several of these secreted enzymes, produced in a large-scale submerged fermentation, have been widely used in the food and beverage industry for decades (Wu *et al.*, 2006). Example of species are *Aspergillus flavus* (Kranthi *et al.*, 2012; Macchione *et al.*, 2008; Malathi and Chakraborty, 1991), *Aspergillus niger* (O'Donnell *et al.*, 2001; Yang and Lin, 1998), *Aspergillus oryzae* (Ogawa *et al.*, 1995; Vishwanatha *et al.*, 2010a; Vishwanatha *et al.*, 2009). *A. oryzae* has been selected as a non-toxigenic strain, due either to its long history of industrial use or to its evolution. Its safety is also guaranteed by more than a thousand years of use in food fermentation. *A. oryzae* grows on the surface of solid materials such as steamed rice, ground soybean or agricultural byproducts, such as wheat bran, rice bran, bagasse and many other substrates, where amino acids and sugars are initially deficient (Vishwanatha *et al.*, 2009). Various species of *Aspergillus* have been studied in detail for the production of proteases under SSF conditions. Alkaline proteases were reported to be produced by *A. flavus* and *A. oryzae* in SSF system.

*Penicillium species* have a great biotechnological potential for the production of proteases and other enzymes. These include *Penicillium* sp., *P.camemberti*, *P. citrinum*, *P. griseoroseum*, *P. restrictum* and *P. roqueforti*. Species of *Penicillium* attracted the attention for the production of alkaline proteases under SSF and SmF conditions. *Penicillium griseoroseum* and *P. camemberti* are known to produce acid proteases under SmF and SSF conditions. A new strain of *P. griseoroseum* IH-02 produced large quantities of an extracellular acid protease when solid state fermentation was carried out on a substrate containing wheat bran and soybean meal as the sole substrate (Ikram-Ul-Haq and Mukhtar, 2007).

The fungus species, *Mucor pusillus* and *Mucor miehei*, secrete aspartate proteases, also known as mucor rennins, into the medium. The enzymes possess high milk-clotting activity and low proteolytic activity, enabling them to be used as substitutes for rennin in the cheese industry (Andrade *et al.*, 2002). The high thermal stability of mucor rennins turned out to be an undesirable quality, since the residual enzyme activity, after cooking, can spoil the flavor of cheese during the long maturation process (Maheshwari *et al.*, 2000). Recently, production of extracellular proteases by *Mucor circinelloides* using glucose as substrate was reported. The *M. circinelloides* enzyme was stable in pH 5.2 and showed maximum activity at 25 °C (Andrade *et al.*, 2002).

Thermophilic fungi produce hydrolases with important characteristics, such as higher thermostability, optimum activity at higher temperatures and high rates of hydrolysis. Thermostable proteases, that act in the temperature range 65–85 °C for the bioconversions of proteins into aminoacids and peptides, have successful applications in baking, brewing, detergents and the leather industry (Haki and Rakshit, 2003; Merheb *et al.*, 2007). A source of thermostable acid protease was identified on the species *Thermoascus aurantiacus* and *Thermomyces lanuginosus*. The *T. aurantiacus* enzyme was produced in SSF system, containing wheat bran, at 60 °C (Merheb *et al.*, 2007). Similar results, of optimum activity at elevated temperatures, were shown by proteases of the thermophilic fungi *Thermomyces lanuginosus* (70, 50 and 45 °C) (Jensen *et al.*, 2002; Li *et al.*, 1997; Macchione *et al.*, 2008) and *Thermomucor indicae-seudaticae* (70 °C) (Merheb-Dini *et al.*, 2010). Yet, proteases from the specie *Aspergillus oryzae* (Vishwanatha *et al.*, 2010a; Vishwanatha *et al.*, 2009) and from *Penicillium* sp. (Germano *et al.*, 2003) showed optimum activities at lower temperatures, 55 °C and 45 °C, respectively.

## Industrial Application of Proteases

Microbial proteases are the leaders of the industrial enzyme market worldwide and account for approximately 60% of the total enzyme sale in the world (Savitha *et al.*, 2011). The global market for industrial enzymes was estimated at $3.3 billion in 2010, and is expected to reach $4.4 billion by 2015 (Abidi *et al.*, 2011). Among the world sale of industrial enzymes, 75% of these are hydrolytic enzymes, of which two-thirds are proteolytic enzymes (Savitha *et al.*, 2011). The wide specificity of the hydrolytic action of proteases finds an extensive application in different industries, such as food, laundry detergent, leather, pharmaceutical, silk, for recovery of silver from used X-ray films and for waste management, as well as in the structural elucidation of proteins, whereas their synthetic capacities are used for the synthesis of proteins (Abidi *et al.*, 2011; Gupta *et al.*, 2002; Johnvesly and Naik, 2001; Qing *et al.*, 2013; Rao *et al.*, 1998; Savitha *et al.*, 2011). Fungal enzymes are commonly used in industries due to various technical reasons, including the feasibility of obtaining enzymes at a high concentration in the fermentation medium and offer a distinct advantage over the bacterial enzymes in terms of easing the downstream processing (Hajji *et al.*, 2010).

Proteases have a large variety of applications in food industry, and among them, those related to the nutritional and functional value of the products. These include improving the digestibility and sensory quality of food, as well as provide health benefits by reducing allergenic compounds (Tavano, 2013).

The principal applications of proteases in food processing are in brewing, cereal mashing, and beer haze clarification, in the coagulation step in cheese making, in altering the viscoelastic properties of dough in baking and in production of protein hydrolysates (Ward, 2011). The hydrolytic quality of proteases is exploited for degradation of the turbidity complex resulting from protein in fruit juices and alcoholic liquors, the improvement of quality of protein-rich foods, soy protein hydrolysis, gelatin hydrolysis, casein and whey protein hydrolysis, meat protein recovery, and meat tenderization (Kumari *et al.*, 2012; Tomar *et al.*, 2008). The major application of proteases in dairy industry is in the cheese manufacturing, where the primary function of enzymes is to hydrolyze the specific peptide bond to generate casein and macropeptides (Rao *et al.*, 1998). The proteases produced by *Mucor michei* and *Endothia parasitica* are gradually replacing rennin in cheesemaking (Demain and Adrio, 2008). Further, proteases play a prominent role in meat tenderization, especially of beef, as they possess the ability to hydrolyze connective tissue proteins as well as muscle fibre proteins (Kumar and Takagi, 1999). Endo and exoproteinases from *Aspergillus oryzae* have been used to modify wheat gluten in baking processes. The addition of proteases reduces the mixing time of the dough and results in increased loaf volumes (Rao *et al.*, 1998). The alkaline and neutral proteases of fungal origin play an important role in the processing of soy sauce and other soy products. Enzymatic treatment results in soluble hydrolysates with high solubility, good protein yield, and low bitterness. Protein hydrolysates commonly generated from casein, whey protein and soyprotein have applications as constituents of dietetic and health products, in infant formulae, clinical nutrition supplements, beverages targeted at pregnant/lactating women and people allergic to milk proteins, and as flavoring agents (Kumar and Takagi, 1999; Ramamurthy *et al.*, 1991; Rao *et al.*, 1998).

The use of enzymes as detergent additives represents the largest application of industrial enzymes. Proteases in laundry detergents account for approximately 25% of the total worldwide sales of enzymes (Demain and Adrio, 2008). The use of enzymes in detergents formulations enhances the detergents ability to remove tough stains and making the detergent environmentally safe. Nowadays, many laundry-detergent products contain cocktails of enzymes including proteases, amylases, cellulases, and lipases (Hmidet *et al.*, 2009). Alkaline proteases added to laundry detergents enable the release of proteinaceous material from stains. The performance of alkaline protease in detergent is influenced by several factors such as pH and temperature of washing solution as well as detergent composition. Ideally, proteases used in detergent formulations should have high activity and stability within a broad range of pH and temperatures, and should also be compatible with various detergent components along with oxidizing and sequestering (Jaouadi *et al.*, 2008; Kumar and Takagi, 1999; Savitha *et al.*, 2011).

Another industrial process which has received attention is the enzyme aided dehairing of animal hides and skin in the leather industry. Leather-making is a processing industry that has negative implications emanating from the wastes associated with industrial processing. In a tannery, a raw hide is subjected to a series of chemical treatments before tanning and finally converted to finished leather. Alkaline proteases may play a vital role in these treatments by replacing these hazardous chemicals especially involved in soaking, dehairing and bating. Increased usage of enzymes for dehairing and bating not only prevents pollution problems, but also is effective in saving time with better quality leather (Zambare *et al.*, 2011). In addition, studies have demonstrated the successful use of alkaline proteases in leather tanning from *Aspergillus flavus* and *Conidiobolus coronatus* (Laxman *et al.*, 2005; Malathi and Chakraborty, 1991).

In the pharmaceutical and cosmetic industries, proteases might be utilized in the elimination of keratin in acne or psoriasis, elimination of human callus and degradation of keratinized skin, depilation, preparation of vaccine for dermatophytosis therapy, and in the increase of ungual drug delivery (Brandelli *et al.*, 2010; Vignardet *et al.*, 2001). Furthermore, these keratinases can remove the scar and regenerate the epithelia, accelerate healing processes, and might act also in the medicine of trauma (Chao *et al.*, 2007). In cosmetic products, proteases can hydrolyze the peptide bonds of keratin, collagen and elastin of the skin. Enzymes such as papain, bromelain and other proteases have been used on the skin for performing smoothing and peeling. The action of these proteases is related to cell renewal, exercising keratinolytic activity, promoting the removal of dead cells in the epidermis and restore the same (Sim *et al.*, 2000). Additionally, the preparation of elastoterase was applied for the treatment of burns, purulent wounds, carbuncles, furuncles and deep abscesses (Gupta *et al.*, 2002). Collagenolytic proteases have been directly employed in clinical therapy, includes wound healing, treatment of sciatica in herniated intervertebral discs, treatment of retained placenta, and as a pretreatment for enhancing adenovirus-mediated cancer gene therapy (Watanabe, 2004).

## Summary and Perspectives

The use of proteases in different industries has been prevalent for many decades and a number of microbial sources exist for the efficient production of this enzyme. Their vast diversity, specific range of action and property of being active over a very wide range of temperature and pH have attracted the attention of biotechnologists worldwide. Although they are widely distributed in nature, microorganisms are the preferred source of these enzymes in fermentation bioprocesses because of their fast growth rate and also because they can be genetically engineered to generate new enzymes with desirable abilities or simply for enzyme overproduction. The search for new microorganisms that can be used for protease production is a continuous process. Proteases have various applications in major areas of food processing, beverage production, animal nutrition, leather, paper and pulp, textiles, detergents, etc. and with the advent of new frontiers in biotechnology, the spectrum of protease applications has expanded into many new fields such as clinical, medicinal and analytical chemistry.

Considering many industrial applications of protease and the importance to identified stable enzymes, this research group had promising results with protease production by fungi isolated from Brazilian Cerrado soil. Some species of filamentous fungi, such as *Aspergillus, Penicillium* and *Paecylomices* have been identified as great producers of extracellular protease in submerged fermentation. Further studies on the optimized conditions will still be performed and the protease production will be conducted in bioreactors.
